# Checklist on the Quality of the Repetitive Peripheral Magnetic Stimulation (rPMS) Methods in Research: An International Delphi Study

**DOI:** 10.3389/fneur.2022.852848

**Published:** 2022-03-22

**Authors:** Cyril Schneider, Andrea Zangrandi, Nico Sollmann, Michaela Veronika Bonfert, Louis-David Beaulieu, the rPMS Consensus Group

**Affiliations:** ^1^Noninvasive Stimulation Laboratory (NovaStim), Neuroscience Division, Research Center of CHU de Québec-Université Laval, Quebec City, QC, Canada; ^2^Department of Rehabilitation, Faculty of Medicine, Université Laval, Quebec City, QC, Canada; ^3^Department of Diagnostic and Interventional Radiology, University Hospital Ulm, Ulm, Germany; ^4^Department of Diagnostic and Interventional Neuroradiology, School of Medicine, Klinikum rechts der Isar, Technical University of Munich, Munich, Germany; ^5^TUM-Neuroimaging Center, Klinikum rechts der Isar, Technical University of Munich, Munich, Germany; ^6^Department of Radiology and Biomedical Imaging, University of California San Francisco, San Francisco, CA, United States; ^7^Division of Pediatric Neurology and Developmental Medicine and LMU Center for Children With Medical Complexity, Dr. von Hauner Children's Hospital, LMU Hospital, Munich, Germany; ^8^University of Quebec in Chicoutimi, Chicoutimi, QC, Canada

**Keywords:** repetitive peripheral magnetic stimulation, Delphi study, rPMS checklist, non-invasive neurostimulation, peripheral neurostimulation

## Abstract

An increasing number of clinical research studies have used repetitive peripheral magnetic stimulation (rPMS) in recent years to alleviate pain or improve motor function. rPMS is non-invasive, painless, and administrated over peripheral nerve, spinal cord roots, or a muscle using a coil affixed to the skin and connected to a rapid-rate magnetic stimulator. Despite the clinical impact and scientific interest, the methodological inconsistencies or incomplete details and findings between studies could make the rPMS demonstration difficult to replicate. Given the lack of guidelines in rPMS literature, the present study aimed at developing a checklist to improve the quality of rPMS methods in research. An international panel of experts identified among those who had previously published on the topic were enrolled in a two-round web-based Delphi study with the aim of reaching a consensus on the items that should be reported or controlled in any rPMS study. The consensual rPMS checklist obtained comprises 8 subject-related items (e.g., age, sex), 16 methodological items (e.g., coil type, pulse duration), and 11 stimulation protocol items (e.g., paradigm of stimulation, number of pulses). This checklist will contribute to new interventional or exploratory rPMS research to guide researchers or clinicians on the methods to use to test and publish rPMS after-effects. Overall, the checklist will guide the peer-review process on the quality of rPMS methods reported in a publication. Given the dynamic nature of a consensus between international experts, it is expected that future research will affine the checklist.

## Highlights

- A checklist was developed to assess the quality of rPMS methods in interventional or exploratory research
- By means of the Delphi method, an international panel of experts reached a consensus on the rPMS checklist items
- The checklist will help improve the quality of rPMS research, including reliability and reproducibility of stimulation protocols.

## Introduction

Repetitive peripheral magnetic stimulation (rPMS) is a technique used to deliver high-intensity magnetic pulses over spinal nerve roots, peripheral nerves, or muscles (1–4). It is non-invasive and painless with negligible side effects. Over the two last decades, this technique has gained popularity to reduce chronic pain or improve sensorimotor function in physiopathology and its clinical potential is currently being tested in several population (1, 5–24).

Despite a drastic increase of the number of publications listed in major research databases in the last years, the rPMS approach remains scarcely investigated as compared to noninvasive brain stimulation such as transcranial magnetic stimulation (TMS) or other peripheral stimulation techniques, such as transcutaneous electrical stimulation (TES) or transcranial direct current stimulation (tDCS). It is also noteworthy that most rPMS publications were preliminary or explorative in nature, reported single cases or case series (25–29), or small sample-sized (9, 16, 25, 30, 31), and explored different health conditions (22, 32) or different stimulation protocols (31, 33). Most importantly, rPMS studies lack of methodological information, which undermines the possibilities to replicate the findings and interpret data [for a comprehensive review, see (3)].

For example, several methods are used to identify the motor threshold of the peripheral target area and to set the stimulation intensity (i.e., subthreshold vs. suprathreshold stimulation). Most commonly, the muscle contraction threshold or the movement threshold has been used. The former is determined by visual inspection or by palpation of a slight muscle contraction (9, 25, 34, 35). The latter is obviously influenced by limb weight, inertia and position in relation to the gravity, resulting in a lower sensitivity and variability (14, 34–36). The surface electromyography (EMG) signal–which is a more objective outcome measure than the visual inspection or palpation–could be used to assess the motor threshold for rPMS (as it is done routinely for TMS of M1). However, when rPMS is applied close to the EMG electrodes, the artifacts produced contaminate the EMG signals and it remains challenging to determine an EMG response threshold. Nevertheless, despite the fact that stimulation intensity is pivotal for rPMS effects (3), terminology used and description of the methods to determine the motor threshold and the choice of rPMS intensity are sometimes confusing and difficult to understand, and not always reported.

Findings of some clinically important rPMS studies cannot be replicated due to unprecise description of the methods and the different stimulation protocol applied (i.e., continuous vs. intermittent stimulation, pulse shape, coil orientation, direction of the induced current in relation to the nerve axis or muscle fibers) [for a review see (3)]. Further, it is commonly acknowledged that some stimulation trials are needed prior to the intervention itself in order to determine the optimal location and the motor threshold (37). The potential impact of these prior stimuli on M1 excitability has already been suggested in TMS studies (38); however, this has never been addressed in relation to rPMS. The missing information on rPMS methods could plausibly lead to misinterpretation of clinical outcomes published and a misunderstanding of the mechanisms of action underlying the functional improvement. For instance, coil orientation could impact rPMS-driven neuromodulation, as studied by motor evoked potentials (MEP) for TMS over M1 (39–42) or by the amplitude and latency of somatosensory evoked potentials (SEP) (43–46).

The present study aimed at reaching a consensus among an international panel of rPMS experts regarding the items that should be reported or controlled in any interventional or exploratory publication on rPMS after-effects. The Delphi method was applied and the consensual checklist proposed a minimum set of items that may influence rPMS after-effects. Other methodological biases not related to rPMS methods were not considered and should be assessed by other critical appraisal tools. The use of our checklist will improve rPMS study designs, increase the quality of data collection when rPMS is administrated, and reduce reporting biases related to rPMS methods in any publication (47, 48). The checklist will also promote study reproducibility in rPMS and guide the peer-review process for the assessment of the quality of rPMS methods in future publications.

## Methods

### Delphi Approach, Steering Committee, and Panel of Experts

#### Delphi Method

The Delphi method consists of a structured communication between experts within a specific field in order to reach a consensus of opinion on a designated topic by means of a series of items to assess in two rounds or more (48–50). The Delphi process thus reduces the variability of responses between experts who eventually meet a consensus. As compared to other techniques to reach a consensus among a group of experts, the Delphi method has at least two advantages (49, 51): respect of each expert's anonymity, which minimizes the possibility that an individual influences the outcome, and contribution of international experts (consensus worldwide) *via* virtual communication (e.g., *via* email or online questionnaires).

#### Steering Committee, Initial Checklist and International Panel of Experts

**Figure 1:**
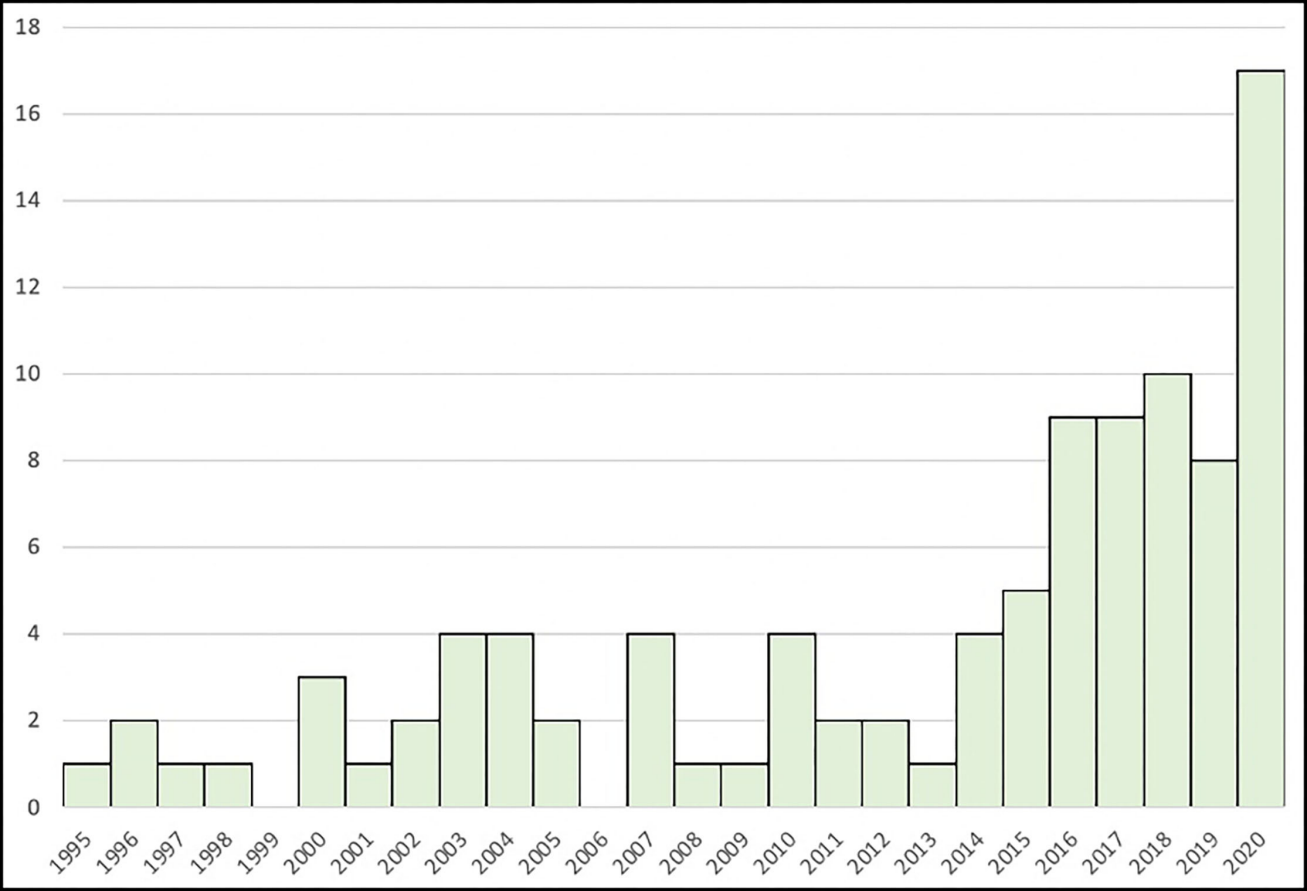
Number of studies using rPMS between 1995 and 2020 and meeting the selection criteria.

The present Delphi-based study was designed and conducted by a steering committee (the first five co-authors whose research has focused on rPMS in the last years). The initial list of items was developed by the members of the steering committee with respect to exhaustive literature and practical experience, as follows: they proposed an initial selection of items based on all rPMS studies published about either clinical administration (e.g., treatment of a health condition) or underlying mechanisms of action (e.g., with healthy people); this list was then enriched with personal experience with rPMS administration (either as researcher or clinician / end-user). Then they had to exchange on the resulting listing until agreement on which items were considered important to be reported or controlled to improve rPMS methods in any future publication. These items were grouped within four domains (subjects, methodology, stimulation protocol, analyses) that were transcribed on a Google form web-based questionnaire. The steering committee had then to identify a group of experts who will have to respond to the questionnaire and reach a consensus on the relevance of the items to be reported or monitored in any rPMS publication. The mandatory criterion to be eligible as an expert was to have already published in the field of rPMS (at least one publication in a peer-reviewed journal as first, senior, or corresponding author) or to be collaborators recommended by the steering committee and with an acknowledged expertise in rPMS. Publications used by the steering committee to develop the initial list of items but also to retrieve rPMS experts worldwide were selected in PubMed and Google Scholar databases. There was no restriction for time (year of publication) or methodological design and only studies on magnetic stimulation at the periphery in humans and published in English were selected by the title, then the abstract and the text. The following keywords were used: “peripheral magnetic stimulation”; “spinal magnetic stimulation”; “magnetic nerve stimulation”; “magnetic muscle stimulation”; “electromagnetic stimulation”. Additional studies were also hand-searched in the references of the selected publications. Figure 1 presents the number of all rPMS publications found per year until 2020.

### Procedures, Instructions, and Web-Based Questionnaire

#### Procedures and Instructions to the Experts

The steering committee first compiled and tested the guidelines and items for univocal clarity plus well-functioning of the web-based questionnaire. The corresponding author then email-invited all eligible rPMS experts worldwide to participate in the Delphi study, the rationale, objective and procedures of the work (two-round, timelines) being detailed and the weblink of the questionnaire appended. Each recipient was blinded to the others to maintain anonymity within the panel, i.e., none of them was aware of the other experts invited to complete the questionnaire and who actually completed it. They were asked to declare potential conflict of interests (CoI) and none was declared. Furthermore, CoI could not interfere because the manuscript did no concern any specific medical devices or indications or applications. Precisely, the steering committee and the experts contributed in two rounds.

In the first round, they had to complete the web-based questionnaire, i.e., to judge each item (that has to be reported or controlled or both) by means of a 5-point Likert scale (different scale for reporting vs. monitoring, see below the section Questionnaire).

In the second round, all experts who had completed the first round were emailed again the weblink of the questionnaire, together with the feedback from the first-round responses in terms of an anonymous percent of responders per item (quantitative data) and the answers from the steering committee to specific comments made (qualitative data). It is noteworthy that the questionnaire used for the second round was identical as for the first round and included the items that were scored weaker after the first round or that did not reach the cut-off threshold chosen for analyzing the results (see details in Data reduction and analysis). This gave experts the possibility to adjust/revise/confirm their responses in the light of the first-round feedback and in order to reach a consensus per item.

Each survey round was open for 2 weeks (after the weblink was emailed) and no participant requested more time. Precisely, a reminder was emailed after the first week of the first round to those who agreed to participate but did not respond yet. A different reminder was sent after the first week of the second round if no response was received yet from those who took part in the first round. Another reminder was sent at the deadline of the second round (2 weeks) in the case of still missing responses from participants to the first round. This strategy was in accordance with a previous work using the same methodology but for a TMS checklist (38).

#### Web-Based Questionnaire

**Figure 2:**
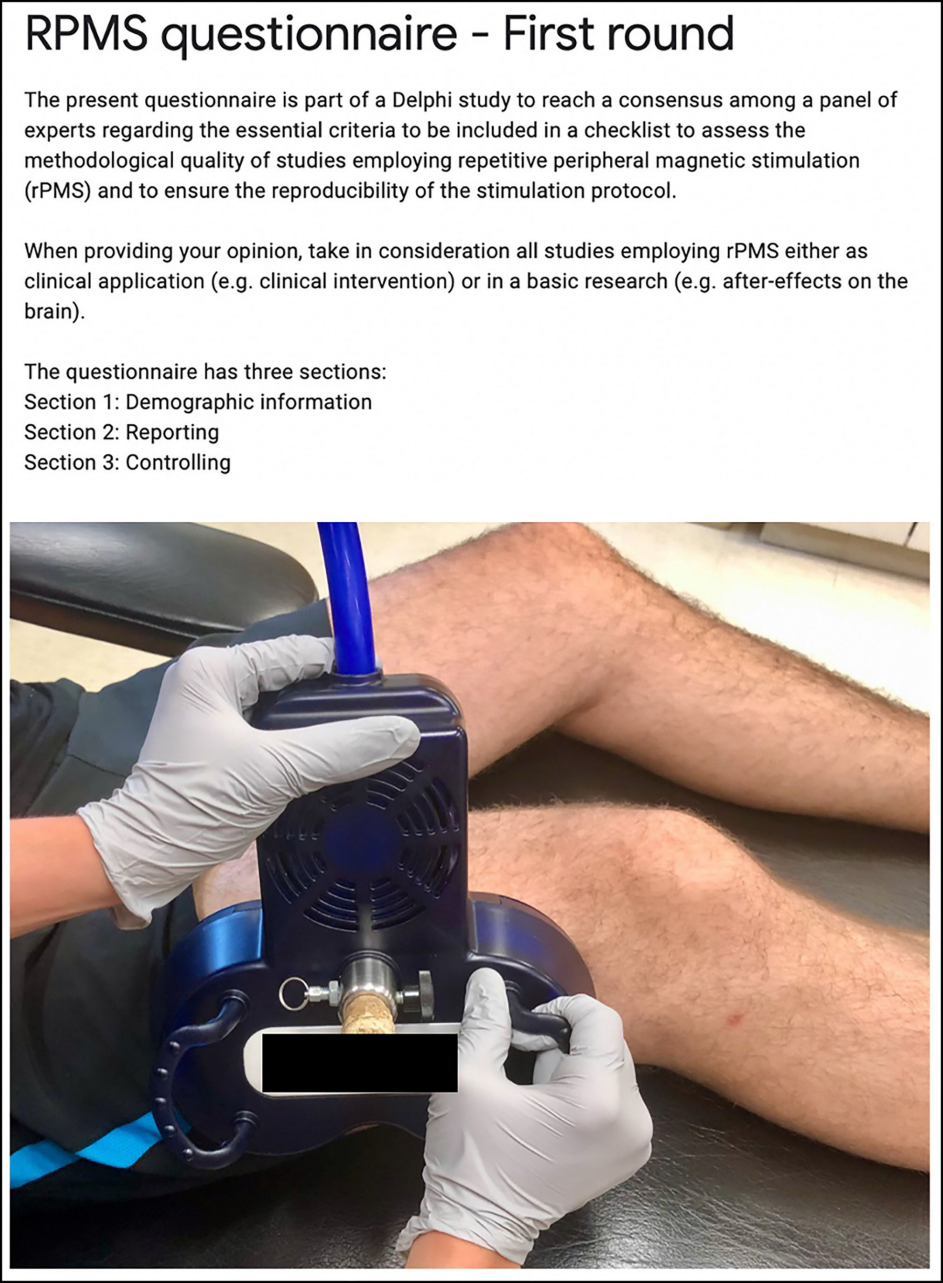
Extract of the first page of the questionnaire for the guidelines to the experts and a custom picture of an example of rPMS administration.

Figure 2 presents the first page of the questionnaire with guidelines and a custom picture for an example of rPMS administration (7-cm outer diameter per wing, biphasic waveform, 400-μs pulse width, device brand anonymized). The questionnaire was structured in three sections.

Section Introduction was used only for the first round to collect demographic information about the experts, including sex, main background, last diploma, place of research work, current affiliation and academic / professional position (see Table 1).

**Table 1:** Characteristics of the international panel of experts for the two rounds.

	***N***	**%**
**Sex**		
Male/Female	14/4	77.8/22.2
**Main background**		
Medicine/Neuroscience / Physiology/Other (e.g., PT, OT)	7/6/1/4	38.9/33.3/5.6/22.2
**Higher-level diploma**		
Master or MSc/PhD/MD	2/12/4	11.1/66.7/22.2
**Place of research work**		
University/Hospital/Research institute	11/6/1	61.1/33.3/5.6
**Current position**		
PhD candidate/*Post-doc*/Researcher/Clinician	2/1/11/4	11.1/5.6/61.1/22.2
**Country** (alphabetical order)		
Canada/China/Germany/Italy/ Japan/Spain	5/1/8/1/2/1	27.8/5.6/44.4/5.6/11.1/5.6

*PT, physical therapy; OT, occupational therapy; MSc, master in science; PhD, philosophy doctorate; MD, medicine doctorate*.

**Figure 3:**
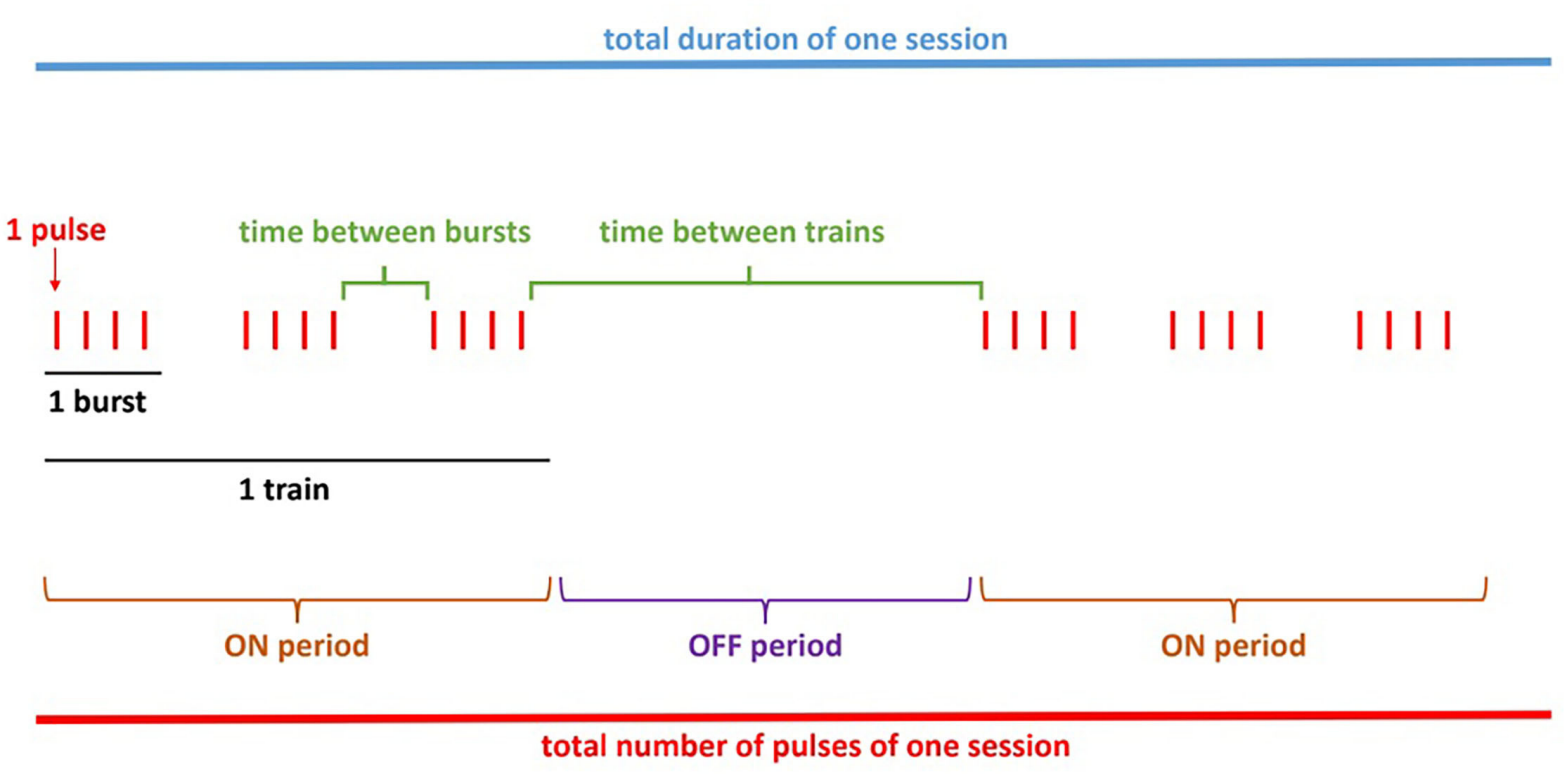
Scheme example of an rPMS stimulation protocol included in the questionnaire. These details aim at avoiding any misunderstanding related to the terminology used.

Section Methods was used to judge whether each item listed should be reported in any rPMS publication (to acknowledge their influence on rPMS outcomes), i.e., as instructed: “*For each item of the list, precise how frequently it should be adequately and clearly reported in a journal publication. Please consider that this concerns any study with rPMS either as clinical application (e.g., clinical intervention) or in basic research (e.g., after-effects on the brain)*”. A total of 46 items were categorized within four domains: subjects (14 items), methodology (19 items), stimulation protocol (11 items), and analyses (2 items). Figure 3 was inserted in the stimulation protocol section to illustrate the terminology and avoid any misunderstanding or doubtful signification of descriptive terms. The 5-point Likert scale used in this “reporting” section proposed the responses “unsure”, “never”, “some of the time”, “most of the time” and “always”, in ascending order with scores from 1 to 5, respectively.

Section Results was used to judge whether the items should be controlled (to minimize their influence on rPMS outcomes), i.e., as instructed: “*For each item of the list, precise how important it would be to control it, either by methodological consistency (e.g., by using the same equipment), research design (e.g., randomization or matching) or by statistical analysis (e.g., as a covariate factor)*.” This “monitoring” section proposed the same items as in the “reporting” section except two items that cannot be controlled (exclusion criteria related to rPMS; side effects of rPMS), thus a total of 44 items within the same four domains: subjects (13 items), methodology (18 items), stimulation protocol (11 items), and analyses (2 items). The 5-point Likert scale used in this “controlling” section proposed the responses “unsure”, “not important”, “somewhat important”, “important”, and “very important”, in ascending order with scores from 1 to 5, respectively.

The webform also enabled the participants to comment on the terminology used or to suggest other items not present in the questionnaire they completed (“*if comments, please specify*”) and, as already mentioned, feedback from the steering committee (qualitative data from the first-round responses) was provided for the second round.

### Data Reduction and Statistical Analysis

Responses from all experts per round, i.e., data collected using the Google Form of the web-based questionnaire, were concatenated into a Microsoft Excel spreadsheet. Three indices were calculated per item: the percent of positive responses, the interquartile range of scores (IQR) and the coefficient of variation of scores (CoV). Statistical analysis was performed with Microsoft Excel Version 16.49 (2021).

The threshold of 75% of “positive” responses (scores 4 or 5 of the Likert scale) was used as a cut-off by the steering committee. This threshold was slightly higher (more severe) than the threshold used in a similar study for a TMS checklist (38). For section Methods, items were rejected (i.e. removed after the second round) if < 75% of the responders chose that they should be reported “most of the time” or “always” (scores 4 and 5 on the Likert scale). For section Results, items were rejected if < 75% of the responders considered them as “important” or “very important” to be controlled (scores 4 and 5 on the Likert scale).

After the second round, the IQR was calculated per item as the absolute value of the difference between the 75th and 25th percentiles of the scores. It is acknowledged that smaller IQR values for an item reflect a higher level of consensus for this item (52). According to previous studies (38, 53), an IQR ≤ 1.00 could be used to select an item for a checklist, in addition to the cut-off related to the percent of positive responses.

The CoV was calculated per item [CoV = standard deviation (SD) / mean score] and a Wilcoxon signed-rank test was used to compare mean CoV between the first and the second rounds. The mean CoV (all items CoV averaged) informed also on the overall variability of responses, i.e., on the general consensus per round.

## Results

### International Panel of Experts

Nineteen (*n* = 19) out of the 43 experts (including the members of the steering committee) who were invited to the survey completed the first round (44.1% responsiveness) and 18 participated to the second round (5.3% attrition). The demographic characteristics of the panel are reported in Table 1. The international panel represented seven countries (all affiliations considered), 66.7% held a PhD, 64% got a position in universities or other research institutes, all were trained in medicine, neuroscience, physiology or rehabilitation related fields (e.g., physical therapy).

### Percent of Positive Responses, IQR, and CoV

In the first round, the cut-off of 75% positive responses was reached for 31 items in the Reporting section and 27 items in the Controlling section. Specifically, all responders scored the items from the “Stimulation protocol” with 4 or 5 points on the Likert scale, thus with an immediate maximal consensus to monitor and control them. A few minor changes of terminology were made before the second round according to comments provided. Also, the net effective depth of stimulation was questioned in relation to two potential additional items to report, namely skin thickness and contact pressure of the coil.

After the second round, the cut-off of 75% positive responses was reached for two more items in the Reporting section (total of 33) and four more items in the Controlling section (total of 31). The IQR of all these items was ≤ 1.00, thus informing on a sufficient level of consensus per item. These data are reported in Table 2.

**Table 2:** Results per item after the second round of the DELPHI study (median scores and IQR).

**Factors**	**Reporting**			**Controlling**		
**Items**	**Median**	**% positive responses**	**IQR**	**Median**	**% positive responses**	**IQR**
***Subjects***						
Age	5	**100%**	0	5	**89%**	0
Sex, gender	5	**100%**	0	5	**83%**	0.75
Handedness or footedness	4	**78%**	1	4	61%	1
Details on the conditions on a participant basis (e.g., specify if affected side was dominant/non dominant previous to the diagnosis)	5	**78%**	1	5	72%	1.75
Previous experience with rPMS (e.g., participation in previous studies)	4	72%	0.75	4	61%	1.75
Prescribed medication and CNS active drugs	5	**100%**	0	5	**83%**	0
Presence/intensity or absence of symptoms related to the health condition (e.g., level of acute pain, level of spasticity, inter-ictal period or migraine)	5	**89%**	0	5	**83%**	0.75
Comorbidity (e.g., presence of neurological/psychiatric disorders)	5	**94%**	0	5	**89%**	0
Exclusion criteria related to rPMS	5	**100%**	0		N/A	N/A
Hormonal/menstrual cycle of female subjects	3	17%	0.75	3	17%	1
Chronic substance intake (e.g., alcohol, drugs, nicotine)	3	50%	1.75	4	56%	2
Substance intake at the day of testing (e.g., alcohol, drugs, nicotine)	4	61%	2	4	61%	2
Habitual physical activity	3	39%	1	3	39%	1
Body Mass Index (BMI)	3	39%	1	3	33%	1
***Methodology***						
Coil location and how optimal placement was defined/reached	5	**100%**	0	5	**94%**	0
Coil type (size and geometry)	5	**100%**	0	5	**94%**	0
Coil orientation	5	**100%**	0	5	**94%**	0
Direction of induced current	5	**94%**	0	5	**89%**	0
Technical specifics of coil (e.g., maximum magnetic field strength, discharge time)	5	**94%**	0	5	**89%**	0
Type of stimulator used (e.g., brand)	5	**100%**	0	5	**94%**	0
Pulse shape (e.g., monophasic or biphasic)	5	**100%**	0	5	**94%**	0
Pulse duration	5	**100%**	0	5	**94%**	0
Cooling used for the coil during stimulation	5	**89%**	1	5	**78%**	1
Use of navigation, if any (e.g., navigation type)	5	**100%**	0	5	**89%**	0.75
Sham procedures, if any	5	**100%**	0	5	**100%**	0
Level of expertise and training of rPMS operator	4	61%	1	4	**78%**	0.75
Number of different operators	4	67%	2	4	72%	1.75
Side effects (e.g., discomfort, pain)	5	**89%**	0	N/A	N/A	N/A
Time of day tested	4	67%	1.75	4	61%	2
Subject attention (level of arousal) during stimulation	3	44%	1	4	**78%**	1
Condition of muscle activity during rPMS (e.g., stimulation with subject at rest or while performing an isometric activity, or subject performing a task)	5	**100%**	0	5	**94%**	0
Presence of hyperalgesia of muscles treated, before and during rPMS (in case of pain disorders)	5	**94%**	0.75	5	**89%**	0.75
Level of relaxation of muscles not stimulated during rPMS	4	61%	2	5	61%	2
***Stimulation protocol***						
Paradigm of stimulation (e.g., continuous, intermittent)	5	**100%**	0	5	**94%**	0
Stimulation frequency	5	**100%**	0	5	**94%**	0
Stimulation intensity	5	**100%**	0	5	**94%**	0
Method for choosing the stimulation intensity (e.g., relation to % of max stimulator output, relation to a sensation/contraction threshold)	5	**100%**	0	5	**94%**	0
Duty cycle (duration of ON and OFF periods for intermittent protocols)	5	**100%**	0	5	**94%**	0
Number of pulses in a burst	5	**94%**	0	5	**89%**	0
Number of bursts in a train	5	**100%**	0	5	**89%**	0
Number of trains in one session	5	**100%**	0	5	**94%**	0
Total number of pulses	5	**94%**	0	5	**94%**	0
Total duration of one session	5	**100%**	0	5	**94%**	0
Total numbers of sessions and time frame (e.g., every second day)	5	**100%**	0	5	**94%**	0
***Analyses***						
Number of stimuli required to determine the optimal location (i.e., potential effects of stimuli already administered prior to the onset of treatment)	4	61%	1	4	61%	1.75
Number of stimuli required to determine stimulation intensity (i.e., potential effects of stimuli already administered prior to the onset of treatment)	4	61%	1	4	61%	1.75

*% positive responses, % responders having scored 4 or 5 on the Likert scale; IQR, interquartile range. Items bolded were scored higher than the cut-off of 75% positive responses*.

**Figure 4:**
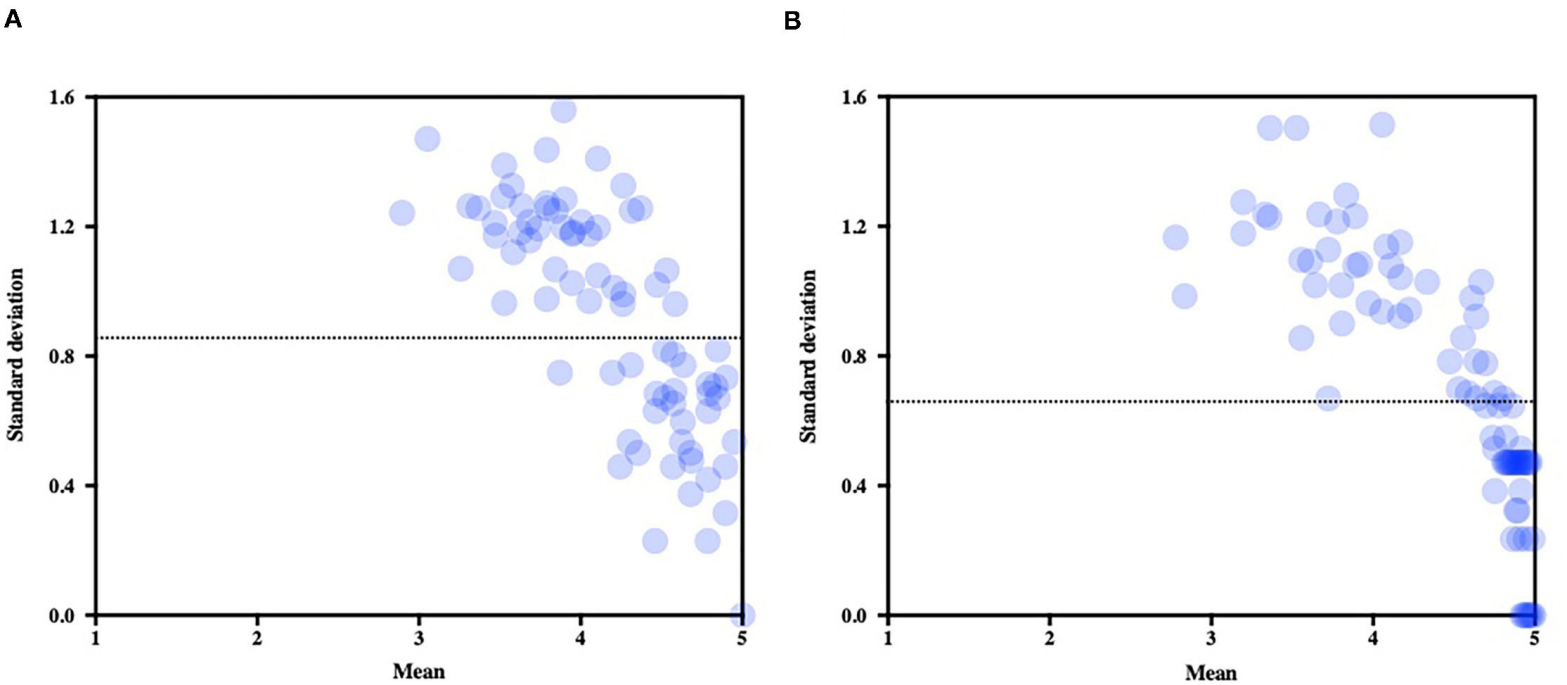
Mean scores (standard deviations expressed against the means) for each item of the questionnaire. Note the decrease of variability after the second round **(B)** as compared to the first round **(A)**. The dotted line represents the average standard deviation in each round.

The mean CoV (average of all items CoV) which informed on the overall level of consensus significantly decreased from the first round (mean CoV = 0.214) to the second (mean CoV = 0.160; *Z* = 514, *p* < 0.001). SD expressed against the mean score of each item score illustrates the decrease of variability between the first round (Figure 4A) and the second (Figure 4B).

### Items Excluded and Final Checklist

Some items did not reach the cut-off of 75% positive responses and were excluded (see Table 3): 2 items in the Reporting section, 2 other items in the Controlling section, and 11 other items in both sections. Thus, these 15 items were not included in the final checklist, which is composed of 8 subject factors, 16 methodological factors, and 11 stimulation protocol factors (see Table 4). A right column titled “Notes” was added in Table 4 to enable comments from the reviewers during the use of the checklist.

**Table 3:** Items excluded from the checklist after the second round of the DELPHI study.

**Section**	**Items**
Excluded only from the “Reporting” section	- Level of expertise and training of rPMS operator - Subject attention (level of arousal) during testing
Excluded only from the “Controlling” section	- Handedness or footedness - Details on the conditions on a participant basis (e.g., specify if affected side was dominant/non-dominant previous to the diagnosis)
Excluded from both sections	- Previous experience with rPMS (e.g., participation in previous studies, etc.) - Hormonal/menstrual cycle of female subjects - Chronic substance intake (e.g., alcohol, drugs, nicotine) - Substance intake at the day of testing (e.g., alcohol, drugs, nicotine) - Habitual physical activity - Body Mass Index (BMI) - Number of different operators - Time of day tested - Level of relaxation of muscles not stimulated during rPMS - Number of stimuli required to determine the optimal location (i.e., potential effects of stimuli already administered prior to the onset of treatment) - Number of stimuli required to determine stimulation intensity (i.e., potential effects of stimuli already administered prior to the onset of treatment)

*Items excluded only from the “Reporting” section reached the cut-off for Controlling only; Items excluded only from the “Controlling” section reached the cut-off for Monitoring only*.

**Table 4:** Consensual checklist obtained after the second round of the DELPHI study.

**Items**	**Reported**	**Controlled**	**Notes**
***Subjects***			
Age	□	□	
Sex, Gender	□	□	
Handedness or footedness	□	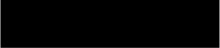	
Details on the conditions on a participant basis (e.g., specify if affected side was dominant/non-dominant previous to the diagnosis)	□	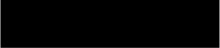	
Prescribed medication and CNS active drugs	□	□	
Presence/intensity or absence of symptoms related to the health condition (e.g., level of acute pain, level of spasticity, inter-ictal period or migraine, etc.)	□	□	
Comorbidity (e.g., presence of neurological/psychiatric disorders)	□	□	
Exclusion criteria related to rPMS	□	N/A	
***Methodology***			
Coil location and how optimal placement was defined/reached	□	□	
Coil type (size and geometry)	□	□	
Coil orientation	□	□	
Direction of induced current	□	□	
Technical specifics of coil (e.g., maximum magnetic field strength, discharge time)	□	□	
Type of stimulator used (e.g., brand)	□	□	
Pulse shape (e.g., monophasic or biphasic)	□	□	
Pulse duration	□	□	
Cooling used for the coil during stimulation	□	□	
Use of navigation, if any (e.g., navigation type)	□	□	
Sham procedures, if any	□	□	
Level of expertise and training of rPMS operator	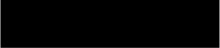	□	
Side effects (e.g., discomfort, pain)	□	N/A	
Subject attention (level of arousal) during testing	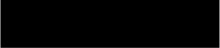	□	
Condition of muscle activity during rPMS (e.g., stimulation with subject at rest or while performing an isometric activity, or subject performing a task)	□	□	
Presence of hyperalgesia of muscles treated, before and during rPMS (in case of pain disorders)	□	□	
***Stimulation protocol***			
Paradigm of stimulation (e.g., continuous, intermittent)	□	□	
Stimulation frequency	□	□	
Stimulation intensity	□	□	
Method for choosing the stimulation intensity (e.g., relation to % of max stimulator output, relation to a sensation/contraction threshold, etc.)	□	□	
Duty cycle (duration of ON and OFF periods for intermittent protocols)	□	□	
Number of pulses in a burst	□	□	
Number of bursts in a train	□	□	
Number of trains in one session	□	□	
Total number of pulses	□	□	
Total duration of one session	□	□	
Total numbers of sessions and time frame (e.g., every second day)	□	□	

## Discussion

The present Delphi study was conducted to reach an international consensus on a checklist for rPMS studies. This checklist ought to represent the minimum dataset of items to be reported or controlled in any interventional or exploratory publication dealing with rPMS. The two rounds were sufficient to reach a consensus on the majority of items, as already suggested (49). The sample of experts obtained (*n* = 19 for the first round; *n* = 18 for the second, seven different countries) fitted the size of 10 to 50 experts usually recommended for conducting a Delphi study (54, 55).

### Minimum Dataset of Items to Be Reported / Controlled in RPMS Studies

The instructions given to the panel of experts (including members of the steering committee) were to provide an opinion for each item listed according to rPMS state-of-the-art and considering the methodological limitations or missing details related to rPMS methods in studies already published. Moreover, the experts were told that the checklist should be the minimum dataset of items to be reported or controlled. Consequently, items that were excluded (due to cut-off of 75% positive responses and IQR > 1.00) are not considered irrelevant and might be included as variables in future rPMS publications. It is indeed conceivable that future research will identify that some new factors can influence the rPMS after-effects or that others do not. This will eventually lead to update the list of factors listed in the present minimum dataset.

For example, the net effective depth of stimulation was questioned after the first round in relation to two potential additional items, one to report (skinfold thickness) and one to report and control (contact pressure of the coil on the skin). The reason was the empiric observation that the efficiency of rPMS of one muscle could be compromised not only by the amount of subcutaneous fat but also by the co-activation of synergistic and antagonistic muscles (e.g., around the knee joint). The latter situation can potentially occur if rPMS intensity is too high or pressure of the coil too strong. Nevertheless, the two potential additional items were not included in the minimum dataset checklist given the challenges to get a standardized measure of skinfold thickness or coil contact pressure. Future publications on rPMS are thus warranted to address this important issue of stimulation efficiency in depth. Similarly, reporting the stimulation intensity in % of maximum stimulator output of the device might not be optimal because this variable, which is not physical, remains difficult to compare between studies that use different stimulators. Rather, the use of the rPMS checklist will help to report more relevant information, such as the motor threshold relative to which the intensity could be expressed, or even the device brand and maximum magnetic field strength, in order to calculate the exact amount of energy delivered.

It is noteworthy that the present checklist focused on the items related to rPMS methods and not to other potential methodological biases (e.g., recruitment, attrition, type of publication, etc.) that could also contribute to variability of results between studies. These other factors could be appropriately assessed by other relevant critical appraisal tools (e.g., (https://casp-uk.net/casp-tools-checklists/) that could be used in combination with our rPMS checklist.

### Consensual Checklist for RPMS Studies

The checklist resulting from the second round's consensus included 35 items in three main domains, namely the subjects (*n* = 8), methodology (*n* = 16), and stimulation protocol (*n* = 11). This checklist is proposed as the minimum dataset to improve the methodological quality of rPMS research. It may be primarily used not only to design an rPMS study, i.e., to properly monitor all items known to potentially impact rPMS after-effects, but also to report the work done as accurately and rigorously as possible. That said, the checklist may guide the peer-review process in assessing the quality of any future study using rPMS. For instance, some factors should be reported while others should be only controlled. Specifically, current evidence on the topic, as it stands, does not provide yet any evidence of an effect of handedness/footedness or side of sensorimotor disorder or pain during an rPMS experiment; however, this should be reported in the manuscript to contribute to the demonstration of a possible impact on rPMS after-effects. Conversely, some items should be monitored for standardization of the methods (and not necessarily reported), as for example the expertise and training of any rPMS operator and the level of a subject's attention/arousal during stimulation. Of note, the number of stimuli required to determine the optimal location of rPMS and the intensity of rPMS (items listed initially in the analyses Section) were excluded from the checklist. As for the TMS checklist published previously (38), these factors were considered to have a potential impact on after-effects, but current evidence and experts of the present Delphi study did not suggest their relevance in the rPMS checklist. Overall, the final checklist warrants, with strong consensus between experts, to report and control items about subjects and related to rPMS application and methodology. This includes items that are rarely reported, such as the direction of the induced current (i.e., coil orientation) or the exact procedure to choose the rPMS intensity. Future research should refine the checklist given the dynamic nature of a consensus between international experts. It is expected also that sham settings will be adapted to the different disorders studied.

### Limitations of the Study

The present checklist should be tested, challenged and its usefulness and relevance confirmed by a few pilot studies before being included in upcoming rPMS publications. Also, given rPMS is a relatively newly emerging expensive technique in basic and clinical research, it is still mainly used in laboratories of most technologically advanced countries, thus the geographical distribution of experts was unbalanced, with a lack of representatives from low- and middle-income countries.

## Conclusion

Our two-round Delphi study recommends a checklist of 35 items (minimum dataset of items for subjects, methodology, and stimulation protocol) that should be controlled in any rPMS research and reported in any scientific publication about rPMS. The checklist will thus assist researchers to improve methodological quality of rPMS research and will guide the peer-reviewing quality of future work. It is expected that this checklist will be refined in the future owing to new evidence on any parameter impacting rPMS after-effects. For instance, adequate sham settings

## Data Availability Statement

The original contributions presented in the study are included in the article. Further inquiries can directed to the corresponding author.

## Author Contributions

CS and AZ conceived and conducted the study. AZ analyzed the data and wrote the paper. CS revised and edited the manuscript. NS, MB, and L-DB contributed to study design and implementation of the questionnaire. All members of the steering committee and members of the panel of experts (rPMS Consensus Group) fulfilled the questionnaire for the two rounds. All authors approved the final version of the manuscript.

## Conflict of Interest

The authors declare that the research was conducted in the absence of any commercial or financial relationships that could be construed as a potential conflict of interest.

## Publisher's Note

All claims expressed in this article are solely those of the authors and do not necessarily represent those of their affiliated organizations, or those of the publisher, the editors and the reviewers. Any product that may be evaluated in this article, or claim that may be made by its manufacturer, is not guaranteed or endorsed by the publisher.
